# A New Method of Treating Chronic Alcoholism by Chloral Hydrate and Bromide of Potassium

**Published:** 1871-09

**Authors:** F. Bradnack


ART. II. *A New Method of Treating Chronic Alcoholism by Chloral Hydrate and Bromide of Potassium*, by F. BRADNACK, M.D.

---

The primary object of this article is to respectfully call the attention of the profession to what the writer believes to be an original method of treating chronic alcoholism; the method consisting in the use of a combination of unusually large quantities of chloral hydrate and bromide of potassium.

The secondary object is to offer a leaf from my experience in the administration of very large doses of chloral hydrate, as a small contribution towards the elucidation of the unsolved problem of the dose of this drug, especially as the last number of this journal contained some interesting correspondence upon this subject.

I think that both these objects will be best accomplished by the narration (as briefly as is consistent with lucidity) of the following case, which is but one of several I could cite:

In June of this year, Mr. S. G., aged 31 years, a victim of chronic alcoholism, came under my charge. The patient at the time of my seeing him, had for five days been drinking so continuously, that it is no exaggeration to describe him as being saturated with alcohol; it fairly exuded from every pore. According to his account, he had during these five days eaten but very little, and slept less.

Having, on the day on which he applied to me been unable to obtain as much whisky as he thought the necessities of his constitution called for, towards evening symptoms of great nervous irritation and exhaustion, began to manifest themselves, consisting principally of that indiscribable uneasiness, and *malaise*, so characteristic of this stage of chronic alcoholism.

On his retiring to bed at 10 p m., I administered to him thirty (30) grains of chloral hydrate. Never have I witnessed a result so instantaneous and remarkable as followed the administration of this medicine, having been, only just prior to swallowing it, in a state of extreme nervous irritability. No sooner was the medicine in the stomach than the patient dropped *immediately* asleep. This occurring with such unprecedented suddenness, alarmed me, (the using the drug at all being somewhat of an experiment,) and I endeavored to arouse the patient. For a moment I succeeded in doing so, and my questions elicited an attempt to answer; and at this point it became interesting to observe that, though the questions were doubtless, understood, the muscles engaged in phonation, and perhaps the nerves also, were almost entirely unable to respond to the efforts of the will. Words were so partially and imperfectly enunciated as not to be understood; and at the expiration of about one minute all ability to exert muscular power in any part of the body ceased entirely, while the patient lapsed into a perfectly death-like sleep; *so* death-like, that, with the exception of some color in the face, he might easily have been mistaken for a corpse.

After satisfying myself, however, that this appearance was only a simulation of death, and not the reality; for the heart still beat, though very slowly, and respiration was not suspended, though it was nearly imperceptible, I left him. He awoke at eight o'clock on the following morning, describing himself as feeling very well, and very much refreshed.

During the period of sleep, the nervous system seemed in an unusually expeditious manner to have recovered its tone, almost all the bad symptoms of the previous evening having disappeared. The only after-effect of the medicine appeared to be a slight amount of drowsiness, which lasted for an hour or two. These results, let it be remembered, followed the administration of thirty (30) grains of chloral hydrate only.

I now come to speak of the effects of largely-increased doses.

In the latter part of June I was again called to the same patient, then suffering from a six days' debauch. He was now in a very much worse condition than on the previous occasion. Sleep seemed impossible, and a sudden decrease in the amount of alcohol, (he having been unable to procure the expected quantity,) had developed the prodromatic stage of *delirium tremens.* To remain quiet was impossible; and he, therefore, walked the floor continuously, his mind being the scene of various delusions. At this stage I urged him to go to bed, which he agreed to do on the condition of being allowed some liquor. It being impossible to induce him to lie down while refusing this request, I deemed it advisable to comply with it. After giving him the whisky, I prevailed on him to take a "dose of medicine," which dose consisted of ninety [90] grains of chloral hydrate. Under the influence of this, he slept quietly until about 3 A.M., when I administered about 20 grains more; after which he slept until morning. He arose considerably refreshed, and would, I think, have made a good recovery without further medication, but for the fact that, during the day he drank, surreptitiously, a large amount of whisky, so that by 2 P.M. he was quite intoxicated, and by 9 P.M., reaction having come on, in a state of nervous irritability so extreme as to verge upon mania, his mind being the constant prey of delusions. Refusing savagely to go to bed without whisky, I, after useless endeavors to overcome his determination, allowed him as before, about two ounces. This temporarily tranquilized him, and under its influence he consented to go to bed, but he declared himself as utterly unable to go to sleep. I thereupon concluded to try an experiment to see whether or no several hours of sleep could not be obtained, it being evident from a variety of symptoms, together with his actions that mania would probably result if sleep was not speedily procured.

Now, it occurred to me that, from its fully-proved sedative action on the cerebro-spinal system, allied to its demonstrated ability to lessen the amount of blood in the brain, as proved by the experiments of Prof. W. H. Hammond, the bromide of potassium, combined with chloral hydrate, the hypnotic and anodyne powers of which I had previously proved to my satisfaction, might constitute a remedy more potent for good than would be either of the drugs administered alone. Acting on this idea, I prepared and administered them in combination, of which the following is the formula:

℞. Chloral Hydrat. - - - - - - ʒ ij.

Potassii bromid, - - - - gr. LXXX.

Syrupi Simp. - - - - - ℥ j.

Aquæ - - - - - - f ℥ ij ss.

M.

*Ft. haust.*

These enormous doses, 120 grains of chloral hydrate, and 80 grains of bromide of potassium, I administered to my patient, merely dividing the mixture into two parts, and diluting each with a little water, both doses being taken within five minutes. The effects of this prescription were very gratifying The patient slept a most tranquil and unbroken sleep until eleven o'clock the following morning, when he awoke. During the day he was very drowsy, and manifested a certain amount of hebetude of mind; but his appetite returned, the bowels moved freely, the pulse was 70, and the skin moist, while both the drowsiness and hebetude of mind gradually disappeared. I gave no medicine the succeeding night, during the latter part of which he slept tranquilly, and, on the following day declared himself well enough to proceed on a business journey, upon which he started, reaching his destination after four days in good health.

The details of the above case go far, it appears to me, to prove presumptively two points: 1st, that in cases of chronic alcoholism, enormous doses of chloral hydrate are not only tolerated, but are productive of great good; and, 2nd, that a combination of bromide of potassium with chloral hydrate furnishes a simultaneous sedative and hypnotic so excellent as to seem to indicate its use in diseases of this nature.

That I have hit upon the exact proportions most suitable to this combination, it is of course, impossible to say; but further experiments will readily decide this question. For the present, it will be sufficient to have pointed out the good effects of the combination; while it may not perhaps, be indulging an unreasonable expectation to hope that the combination suggested in this article may perhaps be found to be as useful in the treatment of cases of chronic alcoholism, as it is probable the recent suggestion of a combination of bromide of potassium and cannabis indica promises to be in the treatment of certain cases of insanity. At any rate it will do no harm to subject the hypothesis to the decisive test of experiment.

